# Benign self-limiting cystic lesion after lower end radius fracture in a child

**DOI:** 10.4103/0019-5413.45333

**Published:** 2009

**Authors:** Gautam D Talawadekar, Marion Muller, Helmut Zahn

**Affiliations:** Department of Trauma and Orthopedics, William Harvey Hospital, Ashford, Kent

**Keywords:** Benign cyst, torus fracture, distal radius

## Abstract

Post-traumatic cystic lesion is usually found adjacent to a healing torus fracture. It is typically asymptomatic and appears just proximal to the fracture line within the area of subperiosteal new bone formation. We report one such cyst in a 7 year old boy, with a brief review of literature to highlight the occurrence of such benign self limiting cystic lesions of lower end radius fracture.

## INTRODUCTION

Post-traumatic cystic lesions are rare but have been reported in the orthopedic, pediatric, and radiological literature. A postfracture cyst is usually a lipid inclusion cyst, which is radiolucent and may be seen adjacent to a healing torus fracture. Failure to recognize this condition can lead to an expensive diagnostic evaluation and create unnecessary apprehension.^1^ These are typically asymptomatic and appear just proximal to the fracture line within the area of subperiosteal new bone formation. Complete resolution is a rule. We report one such cyst complicating a greenstick fracture is reported, together with a brief review of published reports.

## CASE REPORT

**Figure 1:**
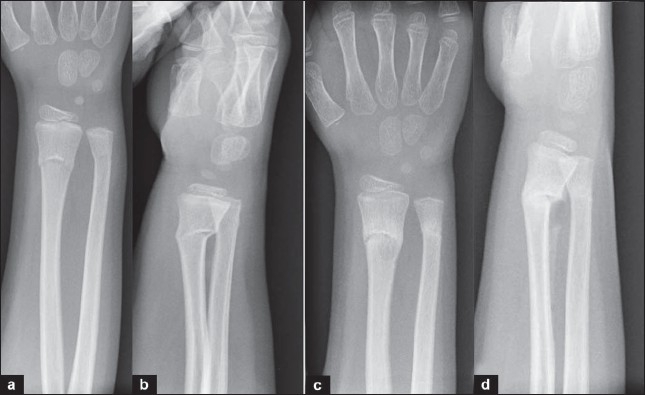
Ateroposterior(a) and lateral(b) X-rays of the forearm with wrist shows Greenstick fracture of the distal radius, with dorsal angulation. Anteroposterior (c) and lateral (d) xray at 3 weeks follow up shows faintly defined cystic area within the subperiosteal callus, just proximal to the fracture site.

A 7-year-old right-hand dominant boy sustained injury to his right wrist following a fall while playing football. The anteroposterior and lateral x-ray of the right forearm revealed a greenstick fracture of distal radius, with dorsal angulation [Figure 1a, Figure 1b)]. The forearm was immobilized in a below-elbow plaster slab that was converted to a cast 3 days later. X-ray examinations at one week showed no change in the alignment. The cast was removed at three weeks. Check x-rays taken at three-week follow-up revealed a faintly defined lucent area within the subperiosteal callus, just proximal to the fracture site [Figure 1c]. The size of the lesion was around 8 mm by 5 mm. The child was comfortable and afebrile, and there were no signs of local inflammation at the fracture site. Differential diagnosis included a preexisting lesion, which was ruled out by the reexamination of the initial x-rays, infection with Brodie abscess formation, or a true postfracture cystic lesion. Whole blood-cell count and C-reactive protein levels were normal. We have decided to leave the child out of plaster and advised to avoid contact sports to prevent reinjury until next follow-up.

**Figure 2:**
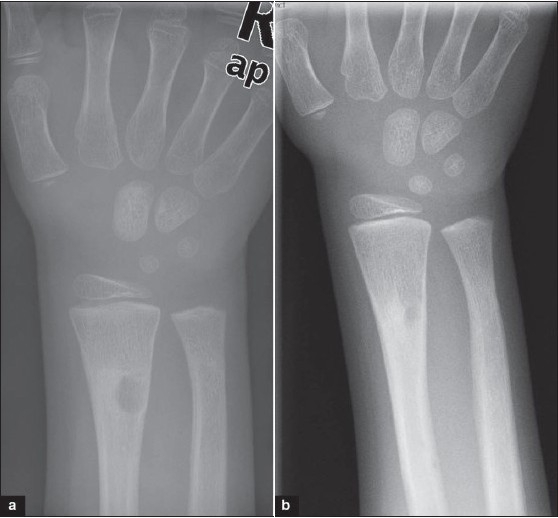
Anteroposterior X-ray (a) of forearm with wrist at 3 month follow-up shows a well defined cystic lesion at the original site of the lesion within the newly formed subperiosteal bone. (b) X-ray at six month follow-up shows complete resolution of the cyst.

**Figure 3:**
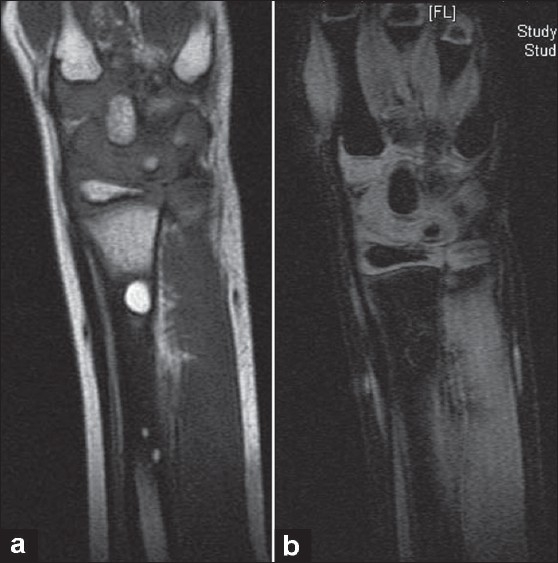
T1WI of MRI (a) at three months follow-up shows area of increased signal with a density similar to that of fat adjacent to the fracture and corresponding to the area of the cystic lesion. Two other coincidental areas of increased signal of unknown significance seen in proximal radial shaft. Fat suppressed image (b) confirming fatty composition of the cyst.

At three months, there was a well-defined 8 mm × 5 mm cystic lesion seen at the original site of lesion within the newly formed subperiosteal bone, proximal to the fracture site [Figure 2a]. The fracture line still remained visible; however, clinically the fracture had united. Magnetic resonance-imaging studies [Figure 3] showed an area of increased signal with a density similar to that of fat adjacent to the fracture and corresponding to the area of the cystic lesion. Two smaller additional coincidental cysts were seen proximally in the radial shaft, away from the fracture site, with density similar to fat, proved on fat suppression images. Significance of these additional cysts remains obscure. There was no clinical suspicion of bone malignancy. Clinically, the child remained asymptomatic, and we have decided to allow him to return to his normal sporting activities and to follow-up at six months. At the six-month follow-up, there was no clinical or radiological evidence of the cyst [Figure 2b] which had completely disappeared, and hence the child was no longer followed up.

## DISCUSSION

Development of a cystic lesion after a fracture is relatively uncommon and is usually seen in children of growing age. Lower end radius fractures seem to be the commonest site of development of these lesions.^1^,^2^ Despite greenstick fracture being the most common fracture of distal part of radius in children,^3^ only 23 postfracture cysts at this site have yet been reported. These postfracture cysts are probably more common than the published reports, because most distal radius fractures in children are monitored on clinical signs alone and their exact incidence is unknown.^4^

These cystic lesions usually appear more than one month after the fracture and characteristically are less than 10 mm in diameter, do not expand, and may be multiple.^4^ In our case, the cyst appeared only three weeks after the fracture, was united and nonexpanding.

There is controversy regarding the etiology of transient postfracture cysts.^1^ Pfister-Goedeke *et al* suggested that they are resorption cysts within an excessive periosteal reaction, related to the subperiosteal hematoma that accompanies greenstick fractures.^5^ Phillips *et al* attribute post-traumatic cysts to the resorption of intraosseous hemorrhage.^6^ The most accepted theory is the intramedullary fat inclusion theory supported by computed tomography description by Malghem *et al*, which proposes that at the time of the fracture, the damaged intramedullary lipocytes release intracellular lipid which passes through the cortical defect and becomes trapped within the subperiosteal hematoma, where it coalesces and is gradually resorbed.^1^,^4^,^7^ MRI findings of the lesion however do not support this theory according to a recent case report by Durr *et al.*^3^ Biopsy of these lesions has never been clinically justified in view of their asymptomatic nature.

The differential diagnosis of cyst-like cortical defects in children included unicameral bone cyst, nonossifying fibroma, an eosinophilic granuloma, osteomyelitis, cystic bone tumors, and transient cyst-like cortical defects.^2^,^8^ Osteomyelitis was excluded in our patient based on clinical absence of signs of inflammation and normal WBC count and CRP. The possibility of a cystic bone tumor was excluded by the supporting history of trauma, the x-ray, and MRI picture, which showed its localization proximal to the site of the fracture, the lack of sclerotic margins of the lesion, and spontaneous disappearance of the lesion.

There is a consensus in the literature regarding the natural history of transient post-traumatic cysts. In most of previously reported cases, the postfracture cortical cysts were asymptomatic. Healing of the initial fracture was not affected by these cysts, nor did they predispose to pathological fractures in any of the reviewed studies. Usually the cyst resolves within two years after the initial injury, and the patient should be managed with observation alone.^1^ In our case, it resolved within six months.

In a review of literature by Davids *et al*, in 1993, the time between the initial fracture and the detection of the lesion ranged from one month to three years and ten months, with a mean of six months in the 18 cases reported at that time in the literature.^1^ The age of the patients in that study ranged from 2½ to 15 years and 15 of the 17 lesions (88.23%) were located in the radius with six patients having multiple cysts (35.29%). In our review, we included five cases reported in English literature since and our own case [Table 1]. The age of the patients ranged from 2½ years to 10 years. All the cases involved the lower end of the radius, with a solitary cyst proximal to the fracture line. Unlike the previous review, one case in our review presented with pain with moderate, soft swelling at the previous fracture site, six months after the fracture.^2^ Hence, the possibility of transient post-traumatic cyst should be included in the differential diagnosis of bone cortical cyst in children even in the presence of clinical symptomatology as x-ray appearance, clinical presentation, patient age, and the time of first diagnosis can vary. In their case studies in 2001, Craig *et al*, have agreed with the most accepted theory of intramedullary fat for the etiology of these cysts, based on CT explanations by Malgham *et al* and MRI explanation by Davids *et al.* However, in 1997, MRI of a post-traumatic cyst by Durr *et al* had excluded the intramedullary fat theory as the signal intensity on both T1 and T2-weighted images exceeded that of fat.

**Table 1:** Review of cases reported in literature since 1993

Series	Age (years)	# location	Initial presentation	Time to appear (weeks)	Time upto disappear (months)
Ball C *et al.*	2.5	L/E radius	Re-fall x-ray	8	N/K
	5.5	L/E radius	Re-fall x-ray	7	15
Garcia-Alvarez F *et al.*	10	L/E radius	Pain, swelling	NK	1
Wass AR	9	L/E radius	Pain after re-fall	NK	NK
Durr *et al.*	6	L/E radius	Incidental at follow-up	6	NK
Our case	7	L/E radius	Incidental at follow-up	3	6

NK: not known; #: Fractures; L/E: lower end

## CONCLUSION

Post-traumatic cystic lesions of the bone remain an under reported entity and the etiology remains unclear. These cysts can present clinically with pain and soft tissue swelling at the previous fracture site, up to six months after the fracture. In all the reported literature, including our own, the cyst disappeared spontaneously.
